# Supplementary material for: Climate change reduces extent of temperate drylands and intensifies drought in deep soils
Source: Nat Commun. 2017 Jan 31;8:14196. doi: 10.1038/ncomms14196 (PMC5290328; doi:10.1038/ncomms14196)
Supplement: Supplementary Information — Supplementary Figures, Supplementary Tables and Supplementary References [file ncomms14196-s1.pdf]

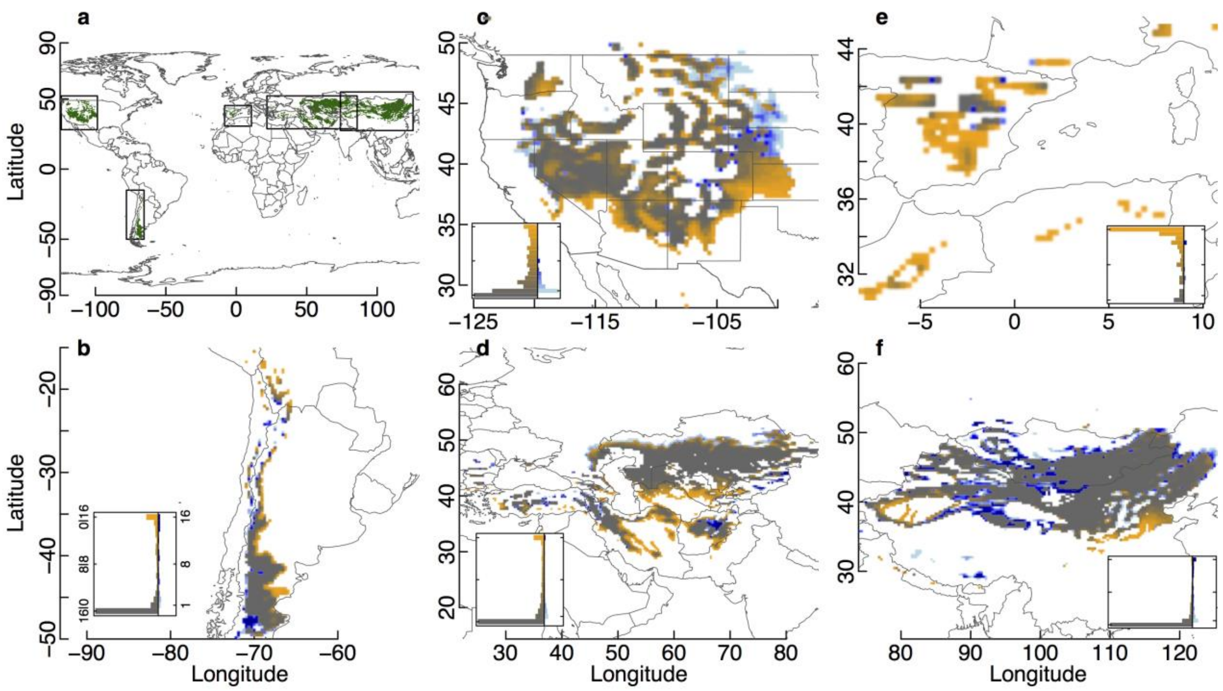

Supplementary Figure 1 | Current and future distribution of temperate drylands. (a) Five temperate dryland regions with their current extent for 1980-2010 (green): (**b**) South America; (**c**) North America; (**d**) Western & Central Asia; (**e**) Mediterranean Basin; (**f**) Eastern Asia. **b-f**, Future projected change in extent under RCP4.5 for 2070-2100, depicted as stable (gray), contracting (orange; no longer temperate dryland in 2070-2100), and expanding (blue; newly temperate dryland in 2070-2100) zones. Inset vertical histograms for panels **b-f** illustrate areal abundance in each category of GCM agreement about expansion or contraction of temperate drylands. Left (gray-orange) histogram depicts GCM agreement (i.e., number of GCMs that agree in the direction of change) about the fate of current temperate drylands and shows the number of cells within each category ranging from pure gray (all 16 GCMs forecast stable temperate drylands) to pure orange (all GCMs forecast conversion from temperate dryland to non-temperate and/or non-dryland). Right (light blue – dark blue) histogram indicates GCM agreement of temperate dryland expansion into new areas and shows the number of cells within each category ranging from dark blue (all GCMs forecast conversion to temperate dryland) to light blue (1 GCM forecasts conversion).

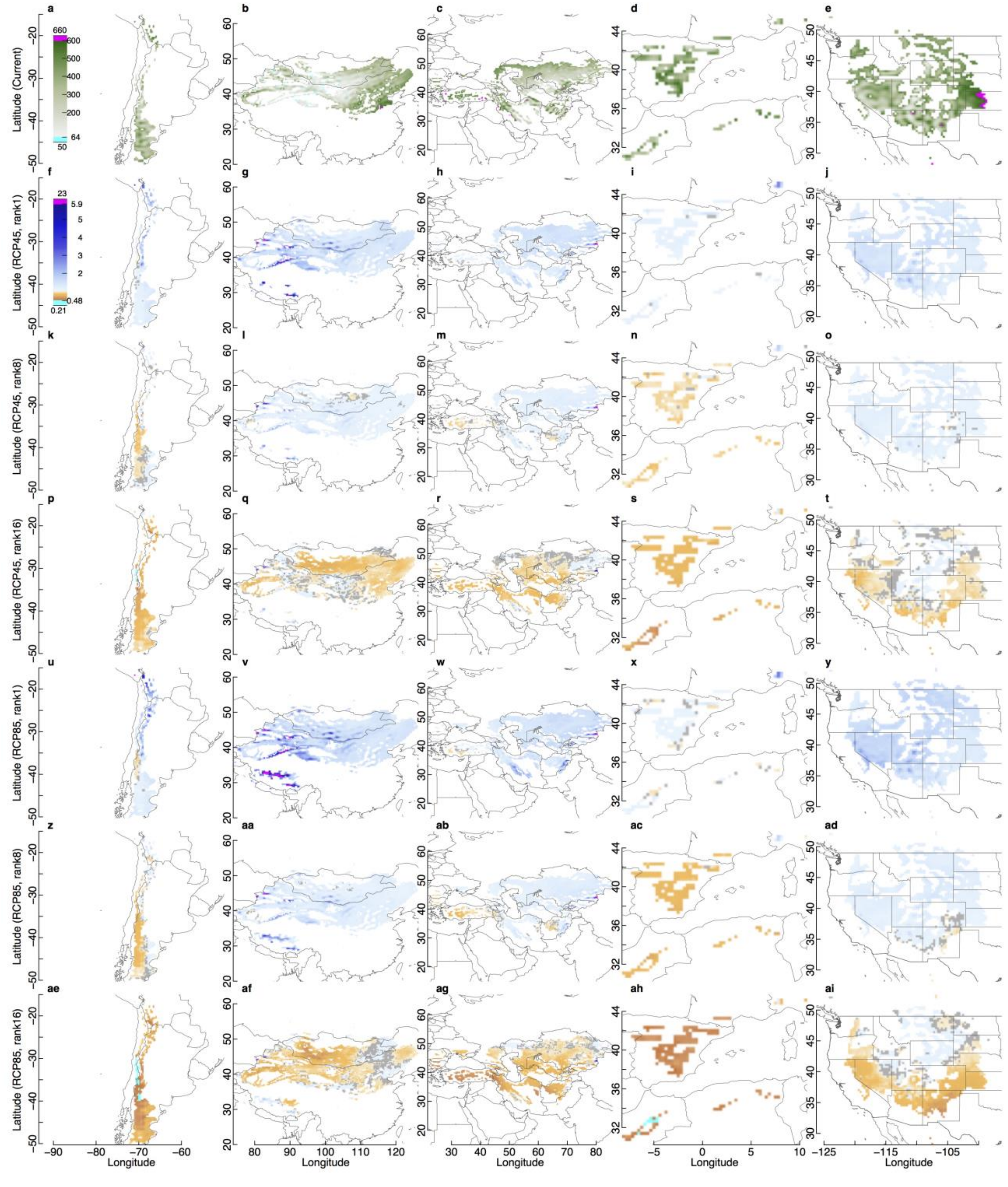

Supplementary Figure 2 | Current climate and scenario conditions. Current mean annual precipitation (*MAP*, mm yr^{-1}) and projected relative changes under future conditions for the end of the 21st century for any cell that is either a current and/or future temperate dryland under any GCM. Ranks refer to the ensemble rank based on 16 GCMs per RCP.

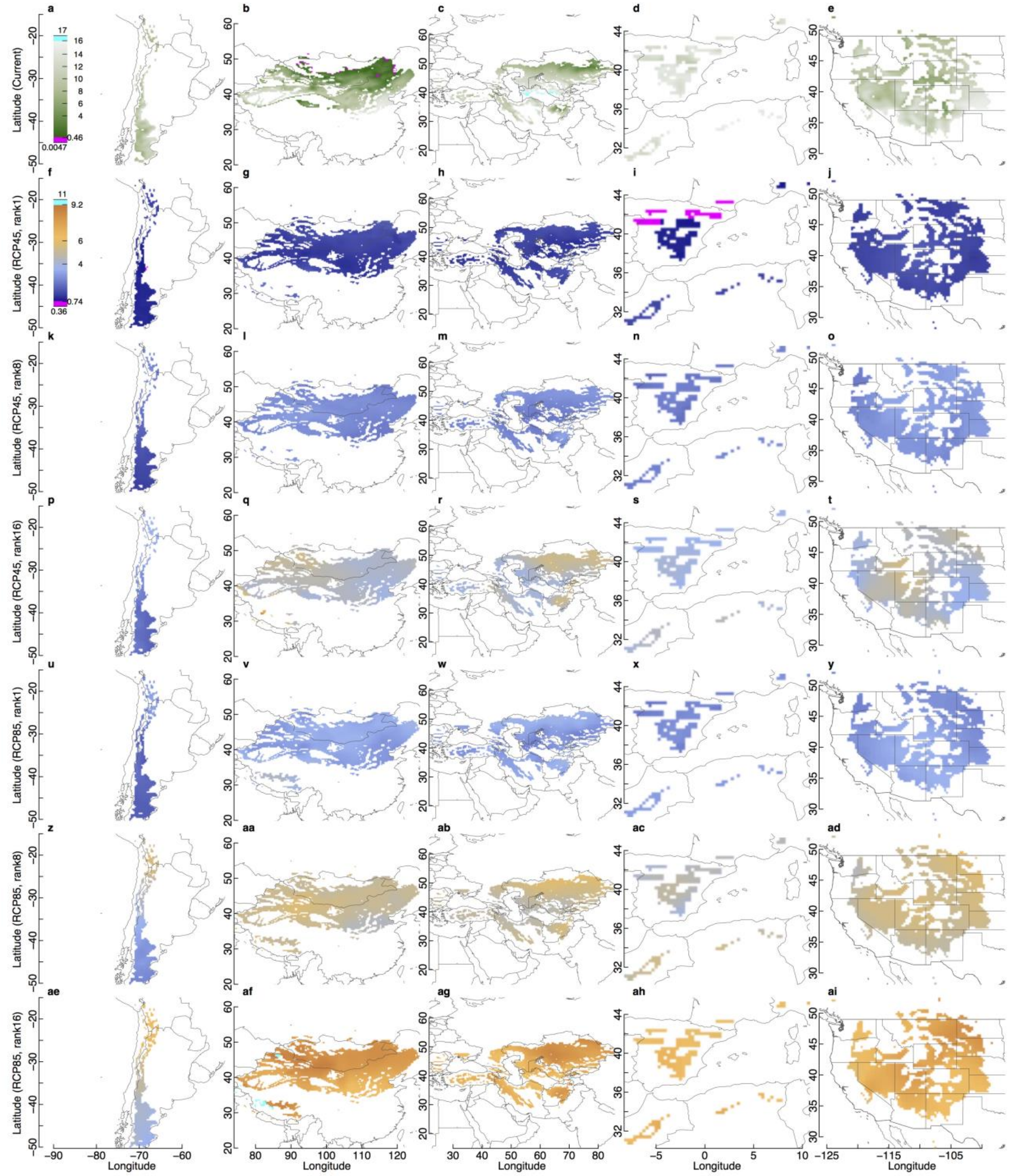

Supplementary Figure 3 | Current climate and scenario conditions. Current mean annual temperature (*MAT*, ˚C) and projected absolute changes under future conditions for the end of the 21st century for any cell that is either a current and/or future temperate dryland under any GCM. Ranks refer to the ensemble rank based on 16 GCMs per RCP.

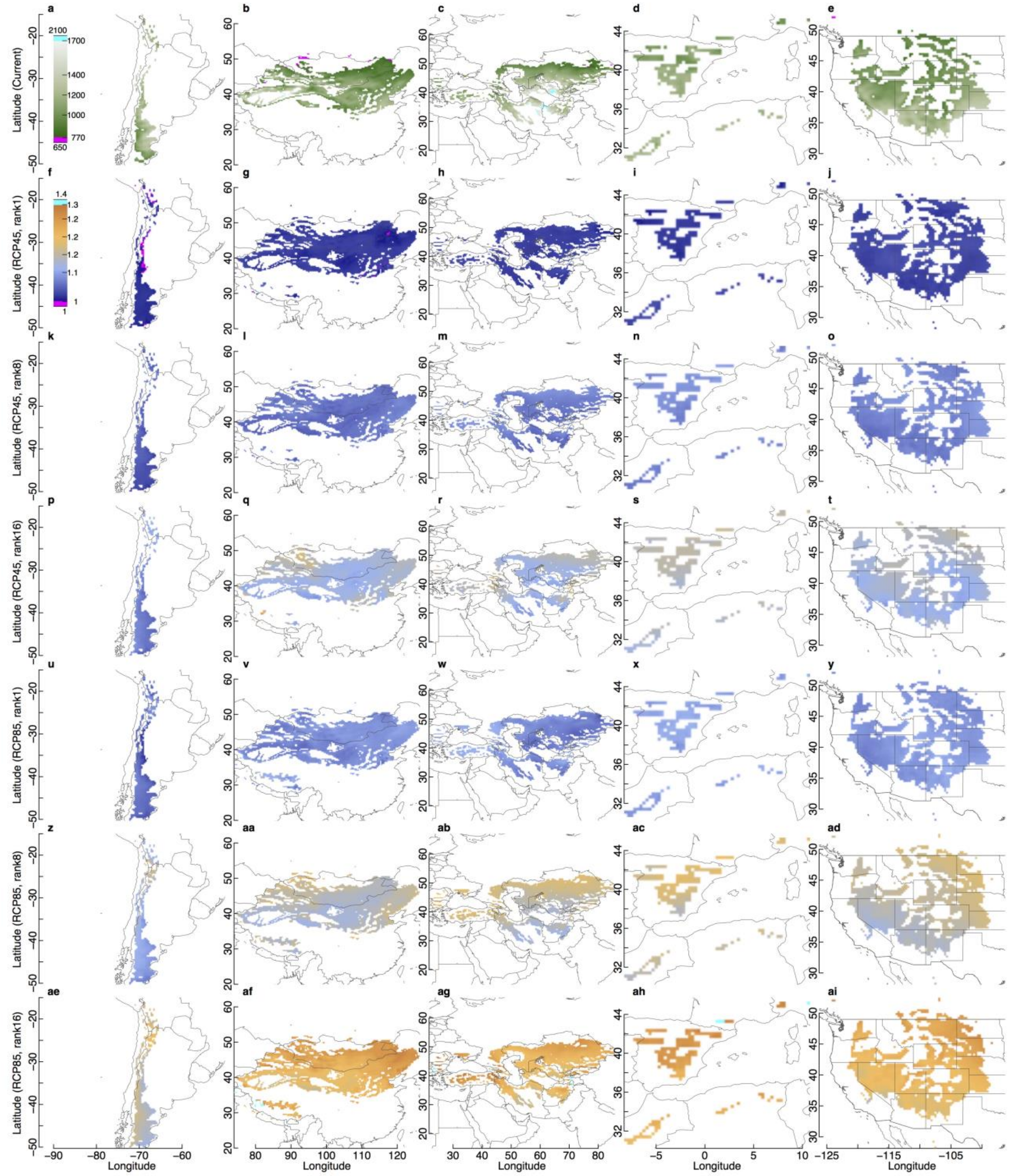

Supplementary Figure 4 | Current climate and scenario conditions. Current mean annual potential evapotranspiration (*PET*, mm yr^{-1}) and projected relative changes under future conditions for the end of the 21st century for any cell that is either a current and/or future temperate dryland under any GCM. Ranks refer to the ensemble rank based on 16 GCMs per RCP.

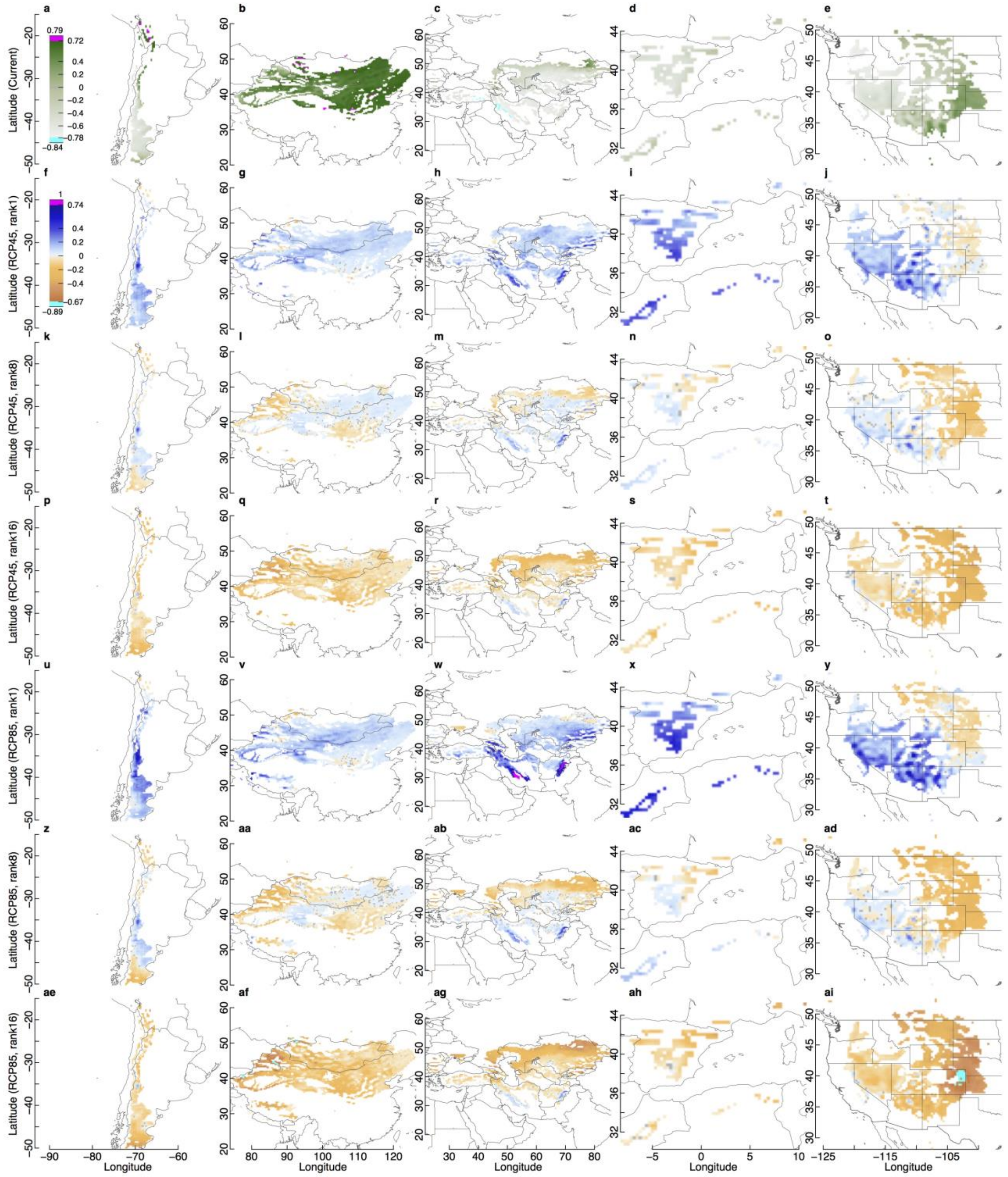

Supplementary Figure 5 | Current climate and scenario conditions. Current mean overlap of wet/warm-season (correlation between monthly precipitation and monthly mean temperature) and projected absolute changes under future conditions for the end of the 21st century for any cell that is either a current and/or future temperate dryland under any GCM. Overlap is the mean annual Pearson correlation coefficient between mean monthly temperature (˚ C) and monthly precipitation (mm). Ranks refer to the ensemble rank based on 16 GCMs per RCP.

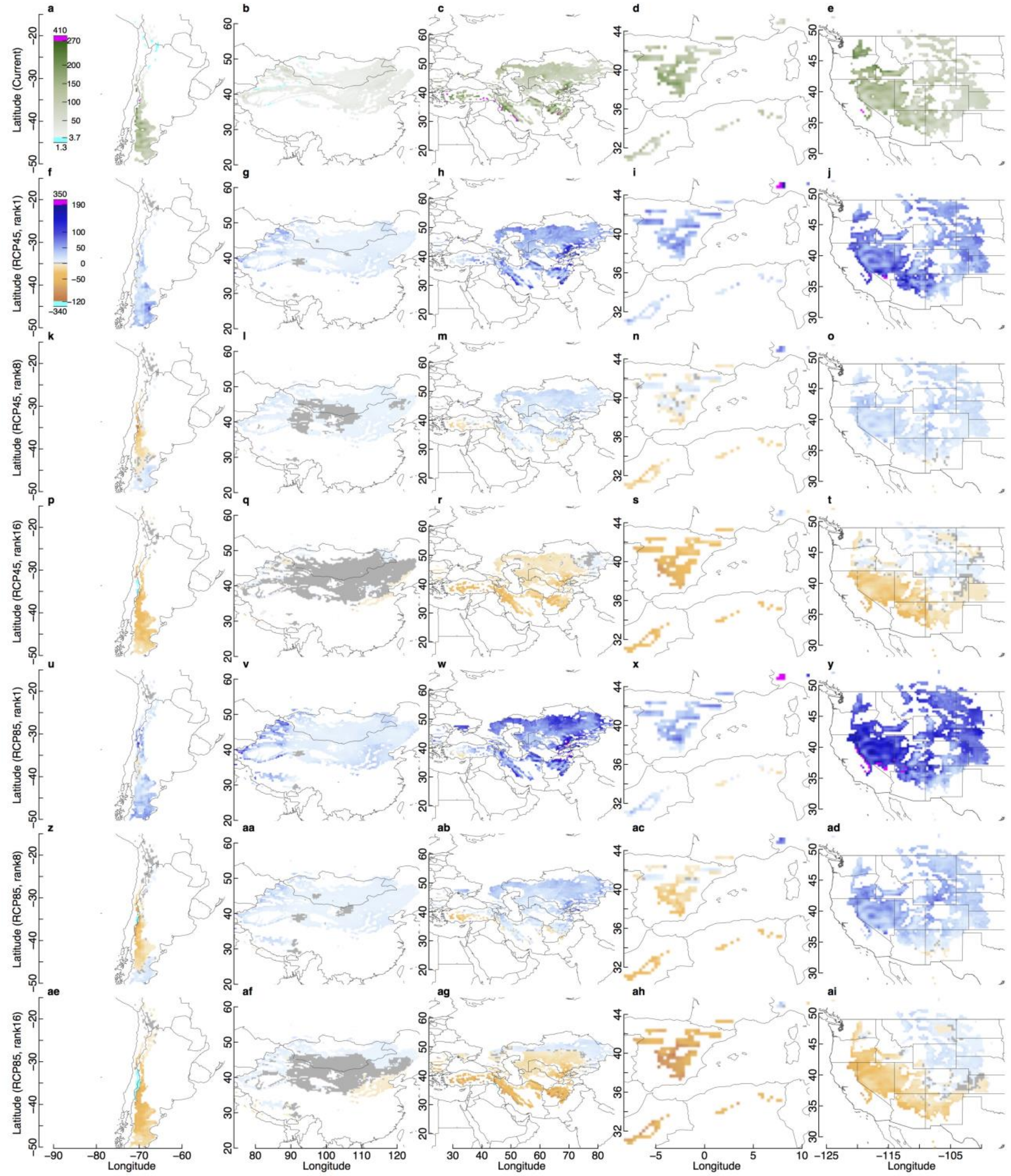

Supplementary Figure 6 | Current climate and scenario conditions. Current mean winter precipitation (mm) and projected absolute changes under future conditions for the end of the 21st century for any cell that is either a current and/or future temperate dryland under any GCM. Winter precipitation is the sum of mean monthly precipitation of December–February on the northern hemisphere, and the sum of mean monthly precipitation of June–August on the southern hemisphere. Ranks refer to the ensemble rank based on 16 GCMs per RCP.

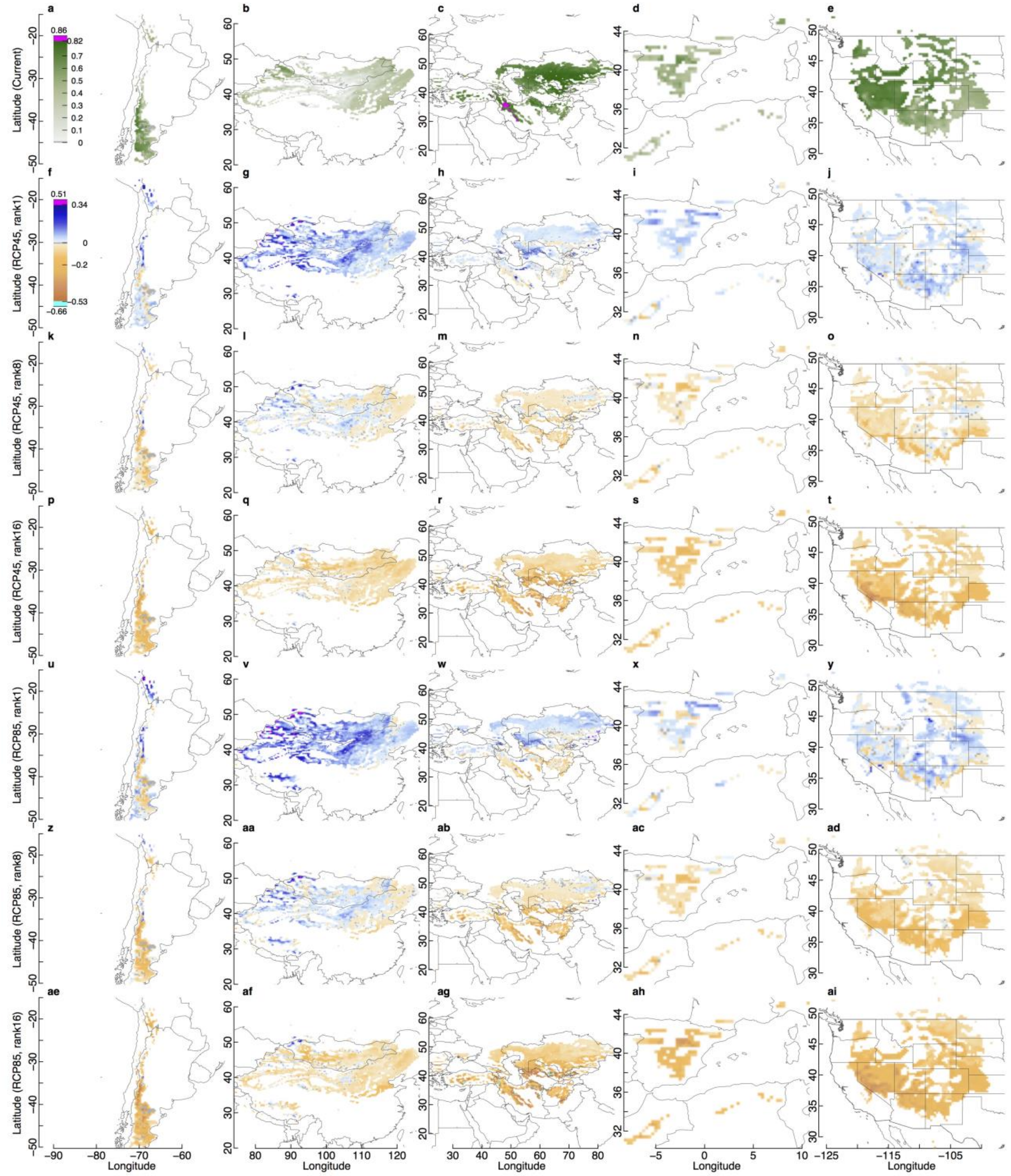

Supplementary Figure 7 | Proportion of transpiration derived from deep soil moisture. Current proportion of transpiration primarily from deep layers, > 20 cm depth, and projected absolute changes in the proportion under future conditions for the end of the 21st century for any cell that is either a current and/or future temperate dryland under any GCM. Ranks refer to the ensemble rank based on 16 GCMs per RCP.

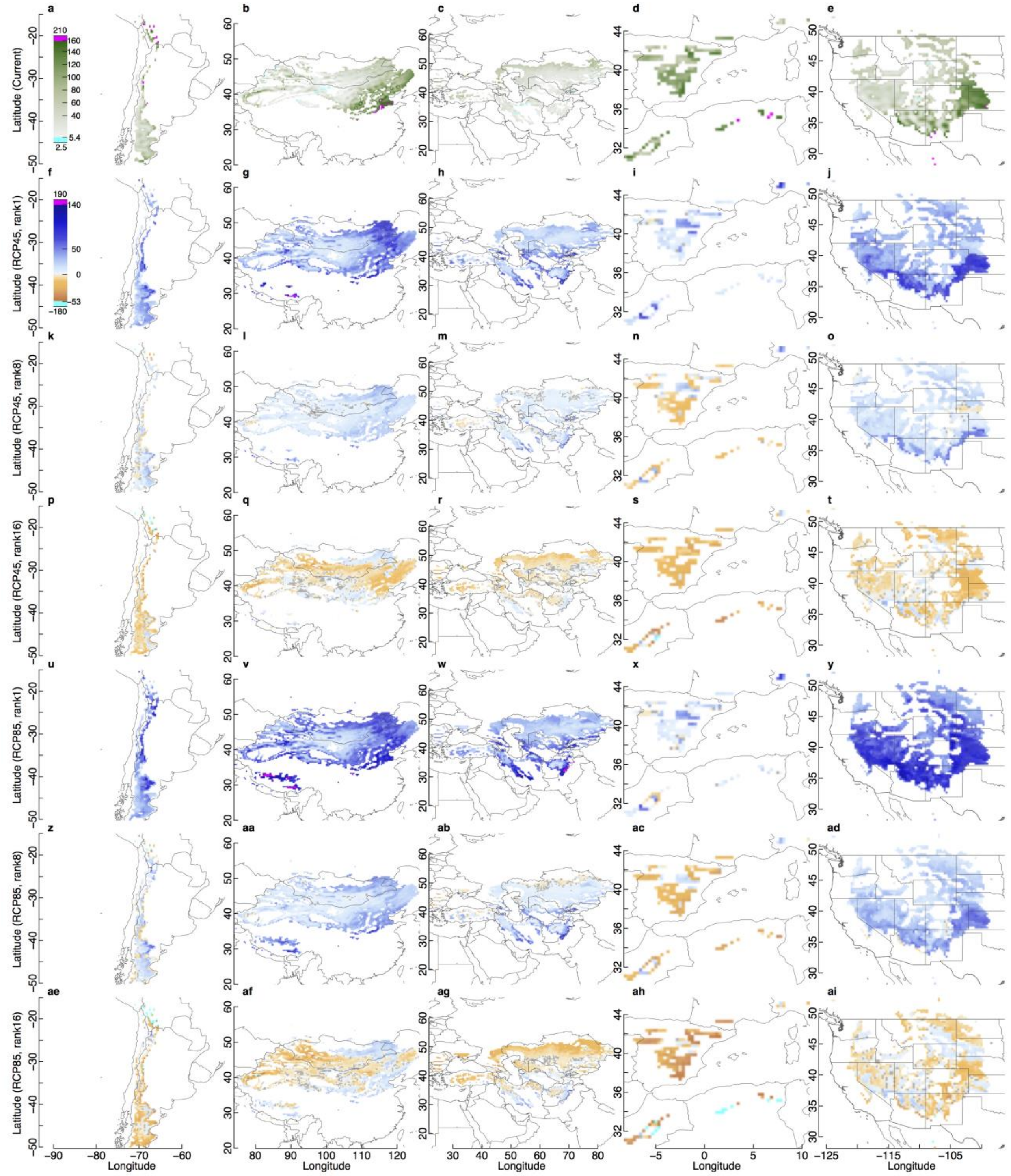

Supplementary Figure 8 | Current and future annual transpiration derived from shallow soil moisture. Current transpiration (mm) from shallow layers, 0–20 cm depth, and projected absolute changes under future conditions for the end of the 21st century for any cell that is either a current and/or future temperate dryland under any GCM. Ranks refer to the ensemble rank based on 16 GCMs per RCP.

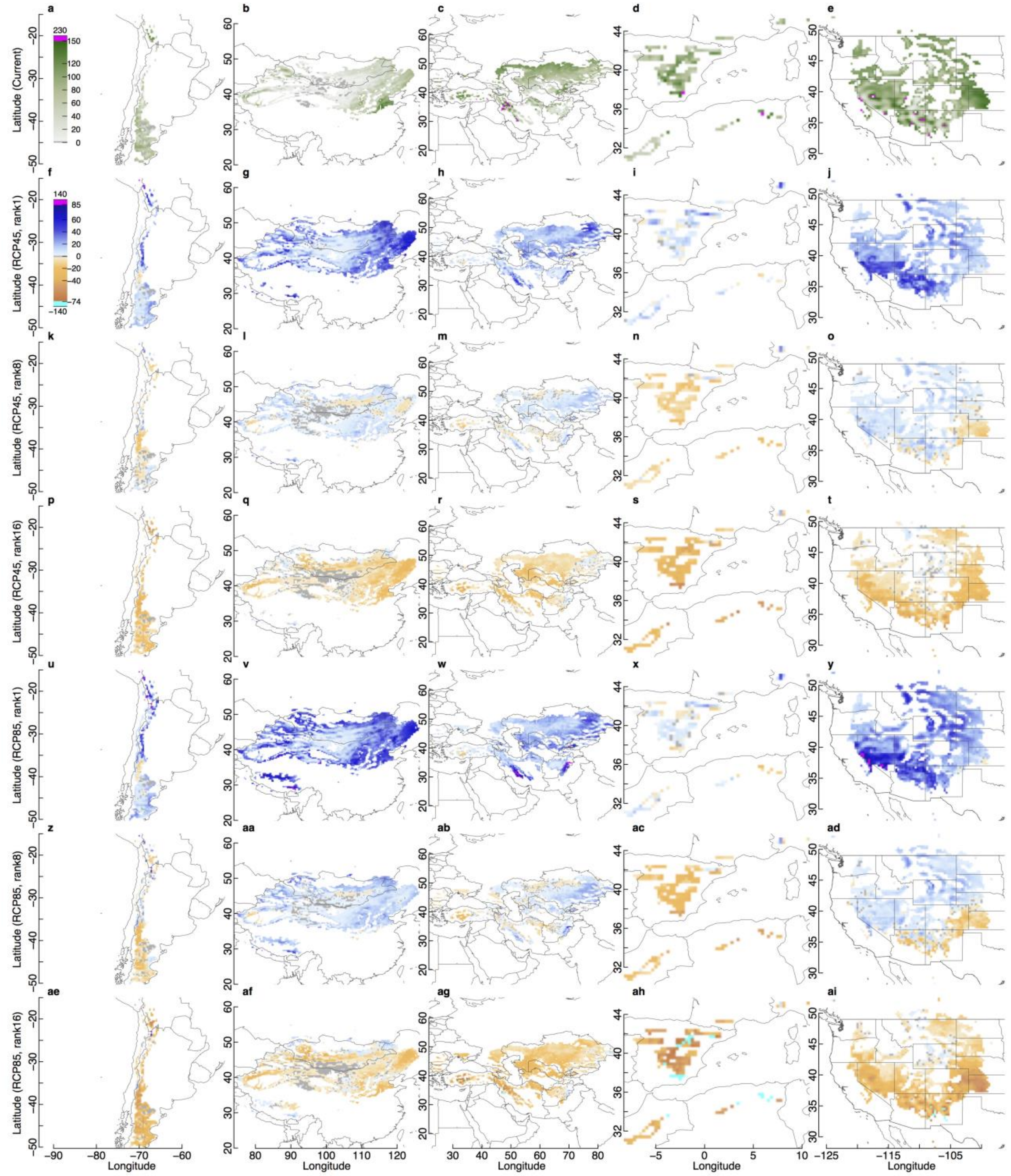

Supplementary Figure 9 | Current and future annual transpiration derived from deep soil moisture. Current transpiration (mm) from deep layers, >20 cm depth, and projected absolute changes under future conditions for the end of the 21st century for any cell that is either a current and/or future temperate dryland under any GCM. Ranks refer to the ensemble rank based on 16 GCMs per RCP.

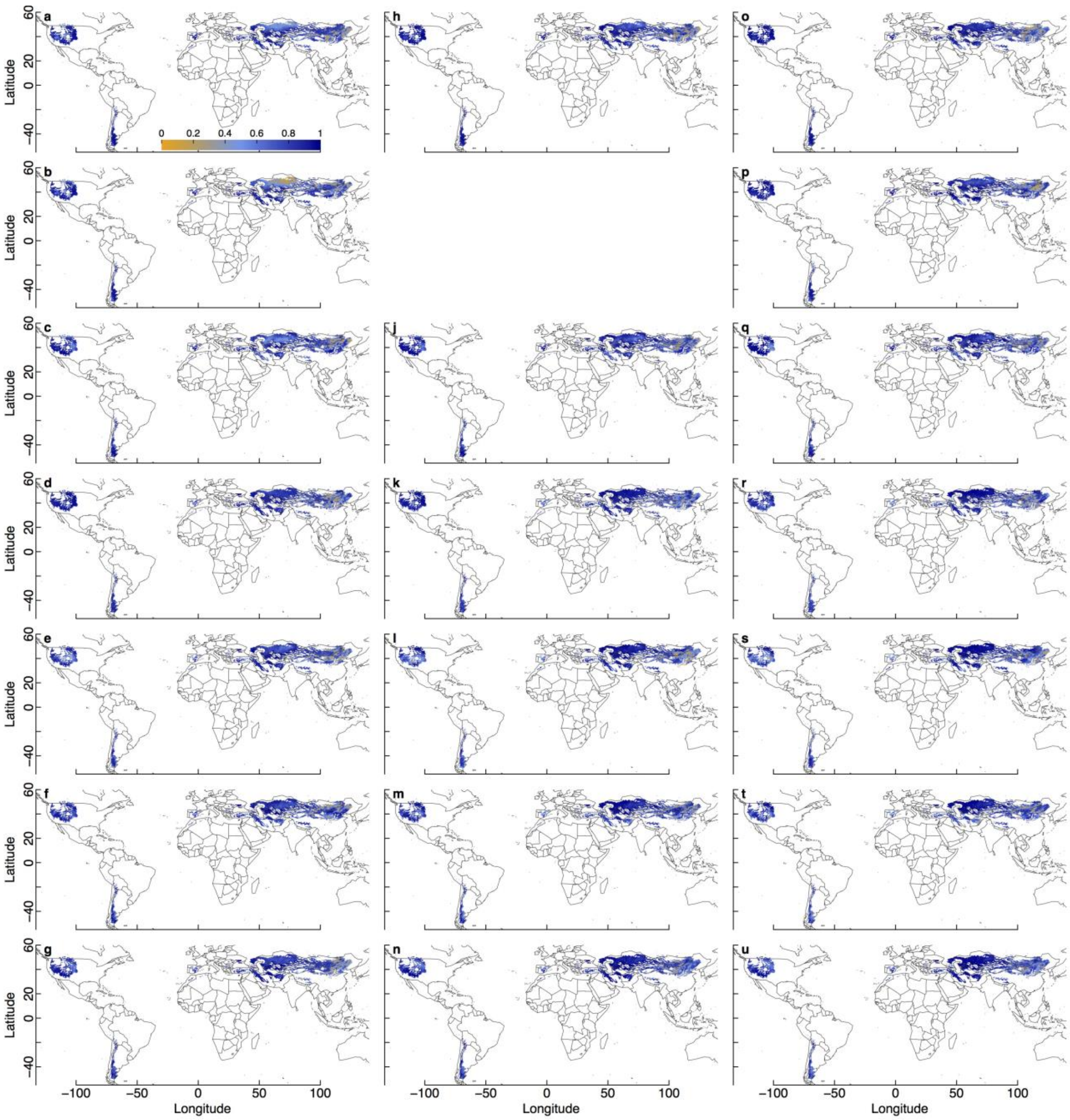

Supplementary Figure 10 | Comparison of mean monthly soil moisture patterns between SOILWAT and GCMs. We calculated mean monthly values of total soil moisture of seven GCMs (FGOALS-g2 (panels **a**, **h**, and **o**); FGOALS-s2 (**b**, **i**, **p**); inmcm4 (**c**, **j**, **q**); IPSL-CM5A-MR (**d**, **k**, **r**); MIROC-ESM (**e**, **l**, **s**); MIROC5 (**f**, **m**, **t**); MPI-ESM-MR (**g**, **n**, **u**)) for the periods of 1980–2005 (**a**–**g**) and 2070–2099 under scenarios RCP4.5 (**h**–**n**) and RCP8.5 (**o**–**u**) for each of our simulated raster cells and estimated agreement with equivalent soil moisture values from SOILWAT output using Duveiller's λ (0 indicates no agreement and 1 is perfect agreement). Note: there are no monthly soil moisture values archived for GCM 'FGOALS-s2' under scenario RCP4.5 (missing panel i).

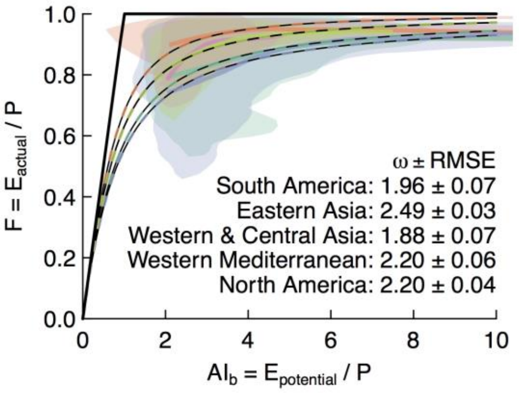

Supplementary Figure 11 | SOILWAT output fitted to the Budyko curve. Illustration of water availability along the Budyko aridity index AI_b (= $1/AI_{UNEP}$) of annual output of SOILWAT for the ratio of actual evapotranspiration (E_{actual}, mm) to annual precipitation (P, mm) c.f. Fig. 1 in ref. [1]. The black line represents the physical demand and supply limits. The Budyko curve parameter was estimated (ω ± RMSE) for each region separately (black/color-dashed lines) based on mean current climate conditions of simulated cells (90% data clouds as shaded areas). Regional output values are summarized by locally weighted polynomial regressions (wide colored lines) and 90% data clouds (shaded areas) for all areas that meet current classification (Fig. 1; turquoise, South America; orange, Eastern Asia; purple, Western & Central Asia; pink, Western Mediterranean; green, North America). Notes: (i) we estimated ω from spatial variation within regions and not from temporal variation within sites; (ii) our simulation study included only temperate drylands, e.g., areas with AI_b < 2 are absent.

Supplementary Table 1 | Distribution of global temperate drylands. Simulated extent (1000 km^2) by region, RCP scenario for the end of the 21st century, level of agreement among GCMs (lowest, middle, highest), and shifting zone.

Scenario	Zone	GCM agreement	South America	Eastern Asia	Western & Central Asia	Western Mediterranean	North America
Current			512	3102	3110	183	1387
RCP4.5							
	Contraction	mid	146	273	769	141	431
		(lo, hi)	(77, 194)	(129, 689)	(479, 1227)	(91, 167)	(109, 857)
	Stable	mid	348	2796	2239	34	953
		(lo, hi)	(317, 435)	(2414, 2973)	(1884, 2631)	(15, 92)	(530, 1278)
	Expansion	mid	40	368	98	3	24
		(lo, hi)	(23, 84)	(254, 433)	(47, 299)	(1, 6)	(2, 135)
RCP8.5							
	Contraction	mid	259	553	1444	173	819
		(lo, hi)	(190, 311)	(356, 956)	(839, 2167)	(144, 183)	(375, 1174)
	Stable	mid	239	2509	1641	10	553
		(lo, hi)	(201, 322)	(2146, 2746)	(943, 2271)	(1, 39)	(213, 1012)
	Contraction	mid	97	466	154	1	40
		(lo, hi)	(50, 169)	(347, 651)	(56, 658)	(1, 4)	(2, 215)

Supplementary Table 2 | Areal change of global temperate drylands. Projected percent decrease, increase, and net change of global temperate dryland area relative to current geographic extent (Supplementary Table 1) by region, RCP scenario for the end of the 21st century, level of agreement among GCMs (lowest, middle, highest), and shifting zone.

Scenario	Zone	GCM agree-ment	South America	Eastern Asia	Western & Central Asia	Western Mediterranean	North America
RCP4.5							
	Contraction	mid	+0.29	+0.09	+0.25	+0.77	+0.31
		(lo, hi)	(+0.15, +0.38)	(+0.04, +0.22)	(+0.15, +0.39)	(+0.5, +0.92)	(+0.08, +0.62)
	Expansion	mid	+0.08	+0.12	+0.03	+0.01	+0.02
		(lo, hi)	(+0.05, +0.16)	(+0.08, +0.14)	(+0.02, +0.10)	(+0.01, +0.03)	(0.00, +0.1)
	Net	mid	-0.19	+0.04	-0.26	-0.79	-0.30
		(lo, hi)	(-0.08, -0.27)	(+0.08, -0.12)	(-0.12, -0.36)	(-0.49, -0.91)	(-0.04, -0.59)
RCP8.5							
	Contraction	mid	+0.51	+0.18	+0.46	+0.94	+0.59
		(lo, hi)	(+0.37, +0.61)	(+0.11, +0.31)	(+0.27, +0.7)	(+0.79, +1)	(+0.27, +0.85)
	Expansion	mid	+0.19	+0.15	+0.05	+0.01	+0.03
		(lo, hi)	(+0.1, +0.33)	(+0.11, +0.21)	(+0.02, +0.21)	(+0.01, +0.02)	(0.00, +0.15)
	Net	mid	-0.31	-0.03	-0.42	-0.93	-0.53
		(lo, hi)	(-0.20, -0.42)	(+0.06, -0.14)	(-0.22, -0.63)	(-0.77, -0.98)	(-0.24, -0.82)

Supplementary Table 3 | Factor attribution to shifts/conversion of temperate dryland distribution. Relative contributions of too low (A^-, AI > 0.5) and too high aridity (A^+, AI < 0.05), temperateness (T, Trewartha's D), and below freezing mean annual temperature (MAT; < 0˚ C) to projected geographic shifts (percent of area of zone type, Supplemental Table 2) of global temperate drylands by region, RCP scenario for the end of the 21st century, GCM-variation (lowest, middle, highest), and zone type. Contributions of more than 35% are highlighted with bold font.

		RCP4.5		RCP8.5	
		Contraction	Expansion	Contraction	Expansion
Region	Attribution	mid (lo, hi)	mid (lo, hi)	mid (lo, hi)	mid (lo, hi)
South America	A-	0.16 (0.02, 0.28)	0.21 (0.04, 0.44)	0.08 (0.01, 0.18)	0.32 (0.20, **0.51**)
	A+	0.01 (0.01, 0.03)	0.05 (0.04, 0.13)	0.02 (0.00, 0.04)	0.04 (0.01, 0.09)
	T	**0.94 (0.84, 1.00)**	**0.88 (0.72, 0.95)**	**1.00 (0.95, 1.00)**	**0.86 (0.75, 0.93)**
	MAT	0.00 (0.00, 0.00)	0.00 (0.00, 0.00)	0.00 (0.00, 0.00)	0.01 (0.01, 0.02)
Eastern Asia	A-	0.28 (0.04, **0.60**)	0.14 (0.01, **0.37**)	0.12 (0.01, **0.36**)	0.15 (0.01, **0.43**)
	A+	0.01 (0.00, 0.17)	**0.37** (0.08, **0.63**)	0.02 (0.00, 0.25)	0.22 (0.01, 0.41)
	T	**0.72 (0.37, 0.92)**	**0.42** (0.31, **0.62**)	**0.88 (0.65, 0.99)**	**0.59 (0.49, 0.69)**
	MAT	0.00 (0.00, 0.00)	0.24 (0.13, **0.41**)	0.00 (0.00, 0.00)	**0.45** (0.30, **0.57**)
Western & Central Asia	A-	0.20 (0.00, **0.45**)	0.50 (0.11, **0.84**)	0.08 (0.03, 0.24)	**0.67** (0.23, **0.92**)
	A+	0.00 (0.00, 0.02)	0.10 (0.02, 0.22)	0.01 (0.00, 0.03)	0.03 (0.01, 0.11)
	T	**0.77 (0.58, 1.00)**	**0.42** (0.18, **0.68**)	**0.93 (0.78, 1.00)**	0.34 (0.11, **0.69**)
	MAT	0.00 (0.00, 0.00)	0.02 (0.01, 0.09)	0.00 (0.00, 0.00)	0.04 (0.02, 0.09)
Western Mediterranean	A-	0.02 (0.01, 0.12)	**1.00 (1.00, 1.00)**	0.02 (0.00, 0.02)	**1.00 (1.00, 1.00)**
	A+	0.00 (0.00, 0.00)	0.00 (0.00, 0.00)	0.00 (0.00, 0.00)	0.00 (0.00, 0.00)
	T	**0.99 (0.88, 1.00)**	0.00 (0.00, 0.00)	**1.00 (0.99, 1.00)**	0.00 (0.00, 0.00)
	MAT	0.00 (0.00, 0.00)	0.00 (0.00, 0.00)	0.00 (0.00, 0.00)	0.00 (0.00, 0.00)
North America	A-	0.31 (0.06, **0.73**)	**1.00 (1.00, 1.00)**	0.16 (0.01, **0.56**)	**1.00 (1.00, 1.00)**
	A+	0.00 (0.00, 0.00)	0.00 (0.00, 0.00)	0.00 (0.00, 0.00)	0.00 (0.00, 0.00)
	T	**0.69** (0.30, **0.94**)	0.01 (0.01, 0.01)	**0.83 (0.47, 0.99)**	0.02 (0.01, 0.05)
	MAT	0.00 (0.00, 0.00)	0.00 (0.00, 0.00)	0.00 (0.00, 0.00)	0.00 (0.00, 0.00)

Notes: (1) Contraction contributions are relative to the extent of the contracting zone, e.g., 100 – 95 = 5% of the area of the contraction will no longer be temperate dryland in South America under RCP4.5 for a medium GCM agreement; similarly, expansion contributions are relative to the extent of the expanding zone, e.g., 91% of the area of the expansion will become temperate drylands in South America under RCP4.5 for a medium GCM agreement. (2) The contributions can sum up to more than 1 because the same area may be affected by several factors simultaneously. Additionally, the sum may be less than 1, particularly at maximum, due to non-linearities of the ensemble calculations, but not in the underlying data at the scenario level (not shown).

Supplementary Table 4 | Simulation responses. Median values of response variables under current and projected future climate conditions for the end of the 21st century and scenario RCP8.5 for shifting zones of each region. Response variables include the mean annual duration of continuous ecological droughts during growing periods for surface soil layers of 0-20 cm depth (DDGP0) and for deep soil layers > 20 cm depth (DDGP20). We calculated DDGP as the longest snow-free, frost-free period when soil water potential (SWP) < -3.0 MPa continuously. We estimated mean annual proportion of transpiration derived from deep soil moisture (> 20 cm depth; T20/T) as the ratio of transpiration resulting from water uptake from deep soil layers (T20) to transpiration resulting from water uptake from all soil layers (T = T0 + T20). Note: range under current conditions is the span from the 0.05 to 0.95 quantiles among cells, i.e., variation within regions, whereas range under future scenario measures variation among GCMs. Areas under current extent correspond to the contracting and stable zone.

Variable	Scenario/ Zone	GCM projection	South America	Eastern Asia	Western & Central Asia	Western Mediterranean	North America
DDGP0	Current		82	50	102	89	61
			(34, 191)	(23, 137)	(39, 203)	(60, 120)	(36, 139)
	Contraction	mid	111	59	141	117	73
		(lo, hi)	(84, 136)	(38, 101)	(123, 168)	(99, 131)	(54, 96)
	Stable	mid	101	49	91	104	75
		(lo, hi)	(84, 127)	(43, 65)	(74, 103)	(82, 117)	(51, 89)
	Expansion	mid	85	44	87	104	54
		(lo, hi)	(59, 101)	(35, 60)	(47, 112)	(85, 124)	(39, 85)
DDGP20	Current		148	118	130	145	95
			(0, 257)	(42, 224)	(62, 233)	(79, 210)	(18, 189)
	Contraction	mid	200	126	190	193	134
		(lo, hi)	(177, 240)	(84, 202)	(182, 226)	(158, 239)	(98, 175)
	Stable	mid	175	126	136	160	123
		(lo, hi)	(158, 210)	(112, 153)	(131, 147)	(141, 184)	(100, 138)
	Expansion	mid	140	111	130	159	94
		(lo, hi)	(125, 152)	(87, 151)	(105, 152)	(137, 172)	(73, 136)
T0	Current		51	44	28	99	55
			(19, 155)	(7, 144)	(9, 76)	(56, 149)	(15, 138)
	Contraction	mid	74.1	106.6	53.6	79	99.1
		(lo, hi)	(49.5, 92)	(41, 151.5)	(35.4, 73.7)	(68.7, 109.1)	(86, 116.7)
	Stable	mid	56.4	57.8	38.8	82.4	67.2
		(lo, hi)	(42.5, 81.9)	(41.9, 71.8)	(29.4, 50.8)	(67.4, 105.8)	(51.1, 97.2)
	Expansion	mid	48.9	58.4	57	79.1	92.4
		(lo, hi)	(41.8, 61.5)	(28.7, 100.2)	(33.8, 77.2)	(53.4, 103.3)	(75.8, 141.1)
T20	Current		58	27	73	78	97
			(0, 103)	(0, 105)	(16, 123)	(39, 147)	(26, 144)
	Contraction	mid	48.5	67.6	58.2	61.2	99.3
		(lo, hi)	(29.5, 64.1)	(32.8, 86.6)	(37.8, 74)	(33.3, 81.6)	(76.3, 118.3)
	Stable	mid	51	33.5	89.8	78.3	105.4
		(lo, hi)	(36.3, 64.6)	(22.1, 40.5)	(70.1, 99.8)	(55.7, 96.1)	(98.5, 114.2)
	Expansion	mid	53.9	25.1	68	61.9	123.3
		(lo, hi)	(34.1, 66.6)	(12.8, 43.4)	(29.5, 112.4)	(58.8, 72.8)	(113.9, 136.1)
T20/T	Current		0.47	0.27	0.69	0.43	0.61
			(0, 0.71)	(0.01, 0.52)	(0.41, 0.81)	(0.28, 0.63)	(0.36, 0.74)
	Contraction	mid	0.33	0.34	0.53	0.39	0.41
		(lo, hi)	(0.25, 0.39)	(0.26, 0.38)	(0.4, 0.59)	(0.27, 0.44)	(0.33, 0.53)
	Stable	mid	0.41	0.27	0.68	0.46	0.6
		(lo, hi)	(0.37, 0.48)	(0.24, 0.28)	(0.65, 0.73)	(0.39, 0.52)	(0.54, 0.66)
	Expansion	mid	0.44	0.25	0.56	0.43	0.55
		(lo, hi)	(0.37, 0.51)	(0.19, 0.27)	(0.49, 0.61)	(0.4, 0.54)	(0.49, 0.64)

Supplementary Table 5 | Simulation responses. Median values of response variables; same as Supplementary Table 4, but under scenario RCP4.5.

Variable	Scenario/ Zone	GCM projection	South America	Eastern Asia	Western & Central Asia	Western Mediterranean	North America
DDGP0	Current		82	50	102	89	61
			(34, 191)	(23, 137)	(39, 203)	(60, 120)	(36, 139)
	Contraction	mid	104	39	148	100	59
		(lo, hi)	(89, 120)	(25, 87)	(112, 170)	(91, 113)	(49, 81)
	Stable	mid	89	49	97	90	71
		(lo, hi)	(80, 100)	(43, 57)	(85, 108)	(81, 104)	(57, 81)
	Expansion	mid	65	52	103	84	44
		(lo, hi)	(47, 81)	(34, 119)	(53, 123)	(69, 101)	(37, 81)
DDGP20	Current		148	118	130	145	95
			(0, 257)	(42, 224)	(62, 233)	(79, 210)	(18, 189)
	Contraction	mid	193	83	188	167	111
		(lo, hi)	(166, 225)	(56, 167)	(151, 214)	(150, 199)	(85, 147)
	Stable	mid	162	125	138	157	114
		(lo, hi)	(150, 174)	(113, 139)	(129, 147)	(140, 175)	(101, 121)
	Expansion	mid	124	128	134	139	80
		(lo, hi)	(112, 132)	(93, 201)	(103, 150)	(105, 167)	(71, 126)
T0	Current		51	44	28	99	55
			(19, 155)	(7, 144)	(9, 76)	(56, 149)	(15, 138)
	Contraction	mid	75.4	126.7	55.6	93.1	95.2
		(lo, hi)	(60.9, 94.4)	(55.9, 150.2)	(31.8, 71.4)	(83.7, 112.8)	(82.5, 106)
	Stable	mid	55.6	52.2	34.1	87.9	61.8
		(lo, hi)	(48.6, 80.4)	(43.3, 62)	(30.5, 40)	(72.4, 104.3)	(52.8, 78.1)
	Expansion	mid	47.6	36.5	39.7	94.4	93
		(lo, hi)	(40.9, 53.4)	(14.6, 63.9)	(28.2, 62.1)	(81, 132.1)	(61.8, 119.4)
T20	Current		58	27	73	78	97
			(0, 103)	(0, 105)	(16, 123)	(39, 147)	(26, 144)
	Contraction	mid	49.5	79.4	56.6	71.3	106.2
		(lo, hi)	(33.3, 67.7)	(43, 93)	(41.6, 92.3)	(53.4, 85.1)	(81, 120.6)
	Stable	mid	58.9	28.4	84.4	73.8	101
		(lo, hi)	(46.4, 67.2)	(22.1, 35.2)	(72.6, 91.9)	(63.5, 90)	(97.4, 108)
	Expansion	mid	60.8	14	38.3	78.2	123.2
		(lo, hi)	(43.5, 75.6)	(3.4, 30.3)	(23.7, 107.3)	(57.6, 132.5)	(110.7, 133.7)
T20/T	Current		0.47	0.27	0.69	0.43	0.61
			(0, 0.71)	(0.01, 0.52)	(0.41, 0.81)	(0.28, 0.63)	(0.36, 0.74)
	Contraction	mid	0.33	0.37	0.56	0.4	0.46
		(lo, hi)	(0.26, 0.38)	(0.31, 0.38)	(0.41, 0.64)	(0.34, 0.45)	(0.37, 0.57)
	Stable	mid	0.44	0.25	0.68	0.44	0.6
		(lo, hi)	(0.39, 0.47)	(0.23, 0.27)	(0.66, 0.72)	(0.37, 0.5)	(0.58, 0.64)
	Expansion	mid	0.52	0.22	0.55	0.45	0.54
		(lo, hi)	(0.41, 0.58)	(0.14, 0.27)	(0.47, 0.61)	(0.37, 0.51)	(0.5, 0.66)

Supplementary Table 6 | Current climate and scenario conditions. Median values of climate drivers under current and projected future climate conditions for the end of the 21st century and scenario RCP8.5 for shifting zones of each region. Note: range under current conditions is the span from the 0.05 to 0.95 quantiles among cells within regions, i.e., variation within regions, whereas range under future scenario measures variation among GCMs. NAs indicate that there were no cells for a shifting zone.

Variable	Scenario/ Zone	GCM projection	South America	Eastern Asia	Western & Central Asia	Western Mediterranean	North America
MAP (mm/yr)	Current		318	253	284	411	393
			(110, 505)	(68, 498)	(127, 541)	(241, 554)	(163, 574)
	Contraction	mid	292	407	287	316	466
		(lo, hi)	(217, 339)	(235, 517)	(211, 350)	(238, 405)	(354, 623)
	Stable	mid	319	286	357	397	446
		(lo, hi)	(241, 365)	(237, 322)	(274, 406)	(309, 497)	(409, 516)
	Expansion	mid	345	296	461	413	529
		(lo, hi)	(307, 384)	(188, 398)	(389, 496)	(374, 525)	(496, 629)
MAT (˚ C)	Current		9.6	6.3	10.6	13.6	10.7
			(6.5, 13.2)	(0.8, 14.2)	(3.9, 15.8)	(10.6, 15.2)	(6, 15.1)
	Contraction	mid	14.5	17.6	18.3	18.2	17.1
		(lo, hi)	(13.6, 15.1)	(16.5, 18.7)	(17.3, 19.1)	(16.7, 19.9)	(15.5, 18.3)
	Stable	mid	11.7	10.8	14.1	14.8	14.1
		(lo, hi)	(11.1, 12.5)	(9.2, 12.6)	(13, 15.8)	(13.8, 15.9)	(13.5, 15.5)
	Expansion	mid	8.7	5.8	11	14.6	14.4
		(lo, hi)	(7.7, 9.5)	(3.9, 8.1)	(7.2, 13.6)	(13.6, 16)	(11.6, 16.5)
PET (mm/yr)	Current		1197	1045	1161	1146	1095
			(934, 1400)	(805, 1414)	(846, 1664)	(979, 1258)	(887, 1370)
	Contraction	mid	1411	1370	1541	1338	1361
		(lo, hi)	(1363, 1449)	(1310, 1435)	(1481, 1575)	(1277, 1412)	(1256, 1400)
	Stable	mid	1263	1167	1224	1140	1178
		(lo, hi)	(1242, 1306)	(1118, 1229)	(1200, 1285)	(1072, 1198)	(1160, 1245)
	Expansion	mid	1114	1003	1093	1128	1137
		(lo, hi)	(1009, 1169)	(943, 1066)	(1004, 1211)	(1081, 1199)	(1061, 1267)
Winter precipitation (mm)[1]	Current		98	18	96	122	93
			(3, 189)	(6, 50)	(48, 231)	(61, 188)	(48, 194)
	Contraction	mid	69	30	111	110	114
		(lo, hi)	(47, 97)	(14, 40)	(86, 141)	(58, 139)	(87, 160)
	Stable	mid	104	22	124	141	135
		(lo, hi)	(93, 132)	(19, 26)	(95, 146)	(102, 180)	(117, 165)
	Expansion	mid	104	20	142	159	111
		(lo, hi)	(81, 122)	(13, 27)	(109, 167)	(123, 209)	(92, 217)
Wet/warm-season overlap[2]	Current		-0.48	0.59	-0.57	-0.46	-0.27
			(-0.66, 0.71)	(0.07, 0.71)	(-0.77, 0.19)	(-0.58, -0.13)	(-0.71, 0.34)
	Contraction	mid	-0.28	0.51	-0.58	-0.46	-0.23
		(lo, hi)	(-0.39, -0.10)	(0.33, 0.65)	(-0.66, -0.46)	(-0.56, -0.12)	(-0.41, -0.09)
	Stable	mid	-0.52	0.58	-0.51	-0.51	-0.47
		(lo, hi)	(-0.63, -0.36)	(0.53, 0.64)	(-0.64, -0.36)	(-0.62, -0.16)	(-0.58, -0.25)
	Expansion	mid	-0.34	0.58	-0.43	-0.56	-0.17
		(lo, hi)	(-0.43, -0.05)	(0.51, 0.63)	(-0.58, -0.10)	(-0.61, -0.47)	(-0.59, 0.083)

[1] Winter precipitation is the sum of mean monthly precipitation of December, January, and February on the northern hemisphere, and the sum of mean monthly precipitation of June, July, and August on the southern hemisphere.

[2] Overlap is the mean annual Pearson correlation coefficient between mean monthly temperature (˚ C) and monthly precipitation (mm). +1 indicates a perfect match between the warm and wet season; -1 indicates a perfect match between the cold and the wet season.

Supplementary Table 7 | Current climate and scenario conditions. Median values of climate drivers; same as Supplementary Table 6 but for RCP4.5.

Variable	Scenario/ Zone	GCM projection	South America	Eastern Asia	Western & Central Asia	Western Mediterranean	North America
MAP (mm/yr)	Current		318	253	284	411	393
			(110, 505)	(68, 498)	(127, 541)	(241, 554)	(163, 574)
	Contraction	mid	293	465	280	376	495
		(lo, hi)	(251, 367)	(295, 525)	(215, 482)	(315, 439)	(409, 562)
	Stable	mid	318	268	330	390	422
		(lo, hi)	(288, 373)	(242, 296)	(285, 359)	(340, 452)	(399, 461)
	Expansion	mid	357	212	438	452	522
		(lo, hi)	(325, 383)	(105, 318)	(305, 464)	(390, 513)	(485, 582)
MAT (˚ C)	Current		9.6	6.3	10.6	13.6	10.7
			(6.5, 13.2)	(0.8, 14.2)	(3.9, 15.8)	(10.6, 15.2)	(6, 15.1)
	Contraction	mid	13.8	15.7	16.9	16.3	15.9
		(lo, hi)	(13.2, 14.2)	(10, 17.2)	(14.9, 17.9)	(15.3, 17.6)	(11.8, 16.7)
	Stable	mid	10.9	8.7	12.8	14	12.9
		(lo, hi)	(10.3, 11.3)	(7.6, 10.4)	(11.6, 14.4)	(13.7, 14.7)	(12.1, 13.9)
	Expansion	mid	7.4	4.9	8.8	13.8	12.8
		(lo, hi)	(6.7, 8.1)	(3.5, 9.1)	(5.1, 9.8)	(12.9, 14.9)	(11.4, 14.4)
PET (mm/yr)	Current		1197	1045	1161	1146	1095
			(934, 1400)	(805, 1414)	(846, 1664)	(979, 1258)	(887, 1370)
	Contraction	mid	1345	1284	1495	1260	1334
		(lo, hi)	(1297, 1383)	(1068, 1362)	(1301, 1563)	(1228, 1314)	(1076, 1376)
	Stable	mid	1238	1112	1212	1114	1153
		(lo, hi)	(1216, 1251)	(1081, 1166)	(1175, 1264)	(1099, 1152)	(1139, 1201)
	Expansion	mid	1009	951	1121	1071	1118
		(lo, hi)	(947, 1121)	(912, 1189)	(929, 1203)	(1019, 1126)	(1027, 1180)
Winter precipitation (mm)	Current		98	18	96	122	93
			(3, 189)	(6, 50)	(48, 231)	(61, 188)	(48, 194)
	Contraction	mid	57	28	111	117	112
		(lo, hi)	(29, 95)	(18, 31)	(87, 142)	(83, 153)	(86, 135)
	Stable	mid	109	20	113	133	114
		(lo, hi)	(83, 130)	(18, 23)	(95, 133)	(91, 157)	(106, 143)
	Expansion	mid	100	15	138	151	100
		(lo, hi)	(87, 121)	(9, 24)	(84, 149)	(105, 193)	(87, 206)
Wet/warm-season overlap	Current		-0.48	0.59	-0.57	-0.46	-0.27
			(-0.66, 0.71)	(0.07, 0.71)	(-0.77, 0.19)	(-0.58, -0.13)	(-0.71, 0.34)
	Contraction	mid	-0.17	0.58	-0.56	-0.44	-0.13
		(lo, hi)	(-0.31, 0.31)	(0.44, 0.68)	(-0.62, -0.43)	(-0.54, -0.2)	(-0.32, -0.00)
	Stable	mid	-0.48	0.6	-0.53	-0.49	-0.36
		(lo, hi)	(-0.57, -0.37)	(0.55, 0.64)	(-0.62, -0.46)	(-0.57, -0.31)	(-0.44, -0.27)
	Expansion	mid	-0.29	0.55	-0.52	-0.52	-0.08
		(lo, hi)	(-0.45, -0.08)	(0.44, 0.63)	(-0.54, -0.02)	(-0.58, -0.24)	(-0.66, 0.18)

Supplementary Table 8 | GCMs used for our simulations. We selected the best performing GCM from each node with available information [2]. CMIP5 GCM available from the "Downscaled CMIP3 and CMIP5 Climate and Hydrology Projections" archive at http://gdo-dcp.ucllnl.org/downscaled_cmip_projections/, their node number in Knutti et al.'s family tree (Fig. 1B in Knutti et al. [3]) after cutting at level 16, and performance against temperature and precipitation observations, i.e., normalized distance from observations (smaller is better; Fig. 3 in Knutti et al. [3]).

CMIP5 GCM	Selection in our study	Node in family tree	Performance
ACCESS1.0		1	0.76
ACCESS1.3		1	0.91
BCC-CSM1.1		2	1.11
BCC-CSM1.1(m)		NA	NA
BNU-ESM		NA	NA
CanESM2	x	10	0.91
CCSM4		11	0.67
CESM1(BGC)		11	0.66
CESM1(CAM5)	x	11	0.60
CMCC-CM		4	0.72
CNRM-CM5		2	0.73
CSIRO-Mk3.6.0	x	14	1.19
EC-EARTH	x	2	0.70
FGOALS-g2	x	15	0.97
FGOALS-s2	x	12	0.96
FIO-ESM		11	0.98
GFDL-CM3	x	5	0.76
GFDL-ESM2G		5	NA
GFDL-ESM2M		NA	0.92
GISS-E2-H-CC		NA	NA
GISS-E2-R	x	7	0.92
GISS-E2-R-CC		NA	NA
HadCM3		NA	NA
HadGEM2-AO		NA	NA
HadGEM2-CC	x	1	0.74
HadGEM2-ES	x	9	0.71
INM-CM4	x	8	1.32
IPSL-CM5A-LR		13	1.01
IPSL-CM5A-MR	x	13	0.96
IPSL-CM5B-LR		NA	1.28
MIROC-ESM	x	16	1.26
MIROC-ESM-CHEM		16	1.27
MIROC4h		NA	0.87
MIROC5	x	3	0.78
MPI-ESM-LR		4	0.71
MPI-ESM-MR	x	4	0.67
MRI-CGCM3	x	6	0.99
NorESM1-M		3	0.87
NorESM1-ME		NA	0.88

Supplementary Table 9 | Variation partitioning as percentages of total variation. Means and standard deviations across variables of climate drivers and of ecohydrological response variables (see 'methods') as mean scenario values and as absolute difference between future scenario conditions and current conditions. Values larger than 5% are highlighted with bold font.

	Climate drivers				Ecohydrological variables			
	Mean values		Differences		Mean values		Differences	
Elements*	Mean	SD	Mean	SD	Mean	SD	Mean	SD
Cells	**68**	**30**	**53**	**36**	**63**	**19**	**68**	**17**
Region	**27**	**31**	**5.2**	3.1	**33**	**20**	**16**	**7**
Shift	2.2	2.4	0.5	0.6	1.2	2.4	0.7	1.0
GCM	1.3	1.2	**20**	**11**	1.1	1.6	**8.6**	**5.1**
RCP	1.3	1.9	**22**	**27**	1.2	2.0	**7.3**	**12**
Region ^ shift	0.8	1.3	0.1	0.2	0.8	0.8	0.3	0.7
Region ^ GCM	-0.2	0.3	-0.1	0.3	-0.1	0.2	-0.1	0.5
Region ^ RCP	-0.3	0.4	-0.1	0.3	-0.2	0.3	-0.4	0.6
Shift ^ GCM	-0.1	0.2	0.2	0.3	-0.1	0.1	0.0	0.3
Shift ^ RCP	-0.2	0.4	0.2	0.4	-0.2	0.3	0.1	0.7
GCM ^ RCP	-0.1	0.1	-0.8	1.1	-0.1	0.1	-0.3	0.5
Region ^ shift ^ GCM	0.0	0.0	-0.1	0.1	0.0	0.0	0.0	0.1
Region ^ shift ^ RCP	0.0	0.1	-0.1	0.1	0.0	0.1	-0.1	0.1
Region ^ GCM ^ RCP	0.0	0.0	0.1	0.1	0.0	0.0	0.0	0.1
Shift ^ GCM ^ RCP	0.0	0.0	0.2	0.2	0.0	0.0	0.1	0.1
Region ^ shift ^ GCM ^ RCP	0.0	0.0	0.0	0.1	0.0	0.0	0.0	0.0

* Entries of elements indicate the uniquely attributable variation as percentage of the total variation. The symbol ^ does not indicate an interaction term, instead it indicates 'substitutability', e.g., 'region ^ shift' indicates that 3.1% of the total variation in mean values of climate drivers are uniquely attributable to 'region' or 'shift', but not to both or to other elements. Negative values indicate that two or more elements together have a higher substitutability than the sum of the uniquely attributable variation by each element individually (see 'methods').

Supplementary Table 10 | Comparison of mean monthly soil moisture patterns between SOILWAT and GCMs. We calculated mean monthly values for the periods of 1980–2005 and projected values for 2070–2099 for each of our simulated raster cells and estimated agreement with equivalent soil moisture values from SOILWAT output using Duveiller's λ [3]; 0 indicates no agreement and 1 is perfect agreement, [4]. Poor agreement (λ ≤ 0.5) is highlighted in italic font; high agreement (λ ≥ 0.9) is highlighted in bold font.

	GCM	GTD	South America	Eastern Asia	Western & Central Asia	Western Mediterranean	North America
Current	FGOALS-g2	0.84	**0.98**	0.61	0.85	**0.99**	0.82
	FGOALS-s2	0.76	**0.96**	*0.49*	0.75	**0.97**	0.82
	inmcm4	**0.94**	**0.96**	*0.37*	**0.90**	**0.96**	**0.93**
	IPSL-CM5A-MR	**0.94**	**0.90**	*0.25*	**0.94**	0.78	**0.96**
	MIROC-ESM	**0.91**	**0.91**	*0.10*	**0.95**	0.68	**0.93**
	MIROC5	**0.91**	**0.92**	0.59	**0.92**	0.79	**0.93**
	MPI-ESM-MR	**0.92**	**0.91**	*0.15*	**0.95**	0.80	**0.92**
RCP45	FGOALS-g2	**0.92**	**0.97**	*0.28*	**0.93**	**0.98**	**0.91**
	FGOALS-s2*	NA	NA	NA	NA	NA	NA
	inmcm4	**0.96**	**0.95**	0.60	**0.94**	**0.96**	**0.95**
	IPSL-CM5A-MR	**0.93**	0.87	0.58	**0.94**	0.74	**0.95**
	MIROC-ESM	**0.90**	0.88	0.65	**0.96**	0.52	0.84
	MIROC5	0.87	**0.90**	*0.30*	**0.90**	0.70	0.88
	MPI-ESM-MR	**0.94**	**0.90**	0.70	**0.97**	0.79	**0.90**
RCP85	FGOALS-g2	**0.95**	**0.97**	0.71	**0.96**	**0.98**	**0.95**
	FGOALS-s2	**0.94**	**0.94**	0.66	**0.94**	**0.99**	**0.96**
	inmcm4	**0.97**	**0.95**	0.68	**0.95**	**0.96**	**0.97**
	IPSL-CM5A-MR	**0.91**	0.83	0.60	**0.95**	0.67	0.89
	MIROC-ESM	0.89	**0.95**	0.72	**0.96**	*0.48*	0.82
	MIROC5	0.86	0.85	0.52	0.88	0.66	0.88
	MPI-ESM-MR	**0.94**	0.85	0.79	**0.97**	0.80	0.88

* There are no monthly soil moisture values archived for GCM 'FGOALS-s2' under scenario RCP4.5

Supplementary References

1 Gudmundsson, L., Greve, P. & Seneviratne, S. I. The sensitivity of water availability to changes in the aridity index and other factors—A probabilistic analysis in the Budyko space. *Geophys. Res. Lett.* **43**, 6985-6994 (2016).

2 Schlaepfer, D. R. *et al.* Simulation of regeneration of big sagebrush supports predicted changes in habitat suitability at the trailing and leading edges of distribution shifts. *Ecosphere* **6**, art3, doi:10.1890/ES14-00208.1 (2015).

3 Knutti, R., Masson, D. & Gettelman, A. Climate model genealogy: Generation CMIP5 and how we got there. *Geophys. Res. Lett.* **40**, 1194-1199 (2013).

4 Duveiller, G., Fasbender, D. & Meroni, M. Revisiting the concept of a symmetric index of agreement for continuous datasets. *Sci. Rep.* **6**, 19401, doi:10.1038/srep19401 (2016).
